# Virtual screening using structure-based consensus pharmacophore models and ensemble docking based on MD-generated conformations

**DOI:** 10.1186/1758-2946-3-S1-O23

**Published:** 2011-04-19

**Authors:** Oliver Koch, Daniel Cappel, Monika Nocker, Timo Jaeger, Leopold Flohé, Christoph Sotriffer, Paul Selzer

**Affiliations:** 1 Intervet Innovation GmbH, Zur Propstei, 55270 Schwabenheim, Germany; 2 MOLISA GmbH, Brenneckestraße 20, 39118 Magdeburg, Germany; 3 Institute of Pharmacy and Food Chemistry, Am Hubland, 97074 Würzburg, Germany; 4 Current Address: Schrödinger GmbH, Dynamostr. 13, 68161 Mannheim, Germany

## 

The protozoan parasites of the genus *Trypanosoma* sp. and *Leishmania* sp. are responsible for neglected diseases like Chagas’ disease, African sleeping sickness or Leishmaniasis. The trypanothione synthetase (TryS) is an attractive new drug target for the development of trypanocidal and antileishmanial drugs [1].

In our virtual screening campaign for targeting the trypanothione synthetase (TryS) we used representative protein conformations derived from a computational analysis using molecular dynamics (MD) simulations of this key component of trypanothione biosynthesis. The publicly available crystal structure lacks a variable loop region that is known to be important for trypanothione biosynthesis. MD simulations turned out to be a good tool to model this loop region and obtain a more complete set of protein conformations for subsequent use in virtual screening [2].

For creating a structure-based consensus pharmacophore model, Superstar [3] was deployed to generate favourable non-bonded interaction maps of different functional groups (probes) for all representative protein conformations. The pharmacophore model was then created for the rigid part of the binding pocket based on high- propensity peaks of these maps. The variable loop region was left out since it can not be depicted by this approach. To include also multiple conformations of the variable loop region, the new ensemble docking feature of Gold was used [4]. After a pharmacophore search within the ZINC database the retrieved molecules were simultaneously docked to the different protein conformations to identify the best combination of ligand pose and protein conformer. Finally, several high-scoring molecules were selected for further testing.

We will discuss in detail this combined pharmacophore/ensemble docking approach based on MD simulations and will present the results of the compound selection for testing.
